# Research in Dermoscopy: The Best Is Yet to Come!

**DOI:** 10.5826/dpc.1101a84

**Published:** 2021-01-29

**Authors:** Aimilios Lallas, Giuseppe Argenziano

**Affiliations:** 1 First Department of Dermatology, School of Medicine, Faculty of Health Sciences, Aristotle University, Thessaloniki, Greece; 2 Dermatology Unit, University of Campania, Naples, Italy

It is not easy to precisely define when dermoscopy was “invented.” The first description of the idea of in-vivo direct skin microscopy goes back to 1950 when Leon Goldman applied it to detect cutaneous filariae [1]. As he extended his research to the in-vivo microscopic examination of nevi, he provided the first pieces of evidence on the potential of this method to uncover clinically invisible morphologic structures of skin tumors [2].

Twenty years later, in 1970, Rona MacKie provided the first description of the microscopic surface patterns of nevi, melanoma, basal cell carcinoma, and angioma [3]. In 1980, Fritsch and Pechlaner improved the technique and suggested that it had the potential of improving the clinical discrimination between benign and malignant skin neoplasms [4].

In the late 1980s the first efforts of systematically categorizing the observed features of lesions and assessing their diagnostic significance were published. Parameters to be evaluated would include patterns, colors, intensity of pigmentation, configuration, regularity, and other characteristics of the surface and the margin of the lesion [5]. This was, in fact, the introduction of pattern analysis in dermoscopy.

A deluge of publications by several research groups followed in the last decade of the twentieth century. Today, most of what is considered basic dermoscopy knowledge sprang from a plethora of publications within that short period. The modified pattern analysis, the ABCD rule of dermoscopy, the Menzies method, and the 7-point checklist were published between 1994 and 1998 [6–9]. Almost simultaneously, large studies on basal cell carcinoma and melanoma on specific locations (eg, acral, face) came to light [10–12].

At the beginning of the new millennium, when the first consensus meeting among dermoscopy experts was held, it seemed that all dermoscopy knowledge had been discovered [13]. It is true that most of the information included in the publication that summarized the consensus meeting of 2000 is still considered valid; but what followed in the increased amount of research, was totally unpredictable.

We used the Scopus database to retrieve data on publications on dermoscopy, using the following search terms: “dermoscopy” OR “dermatoscopy” OR “epiluminescence microscopy.” Our search revealed a total of 17,213 items. Of them, 392 items had been published in the years leading up to and including 2000 and 16,821 items have been published since 2001. Of the latter group, 3,426 were published between 2001 and 2010 and 13,395 between 2011 and 2020. The graphs below illustrate the number of publications per year, highlighting the almost exponential increase (Figures 1, 2, 3).

Several factors might be driving this impressive trend. Improved understanding of dermoscopic morphology generated the need for more profound investigations. The expansion of the use of dermoscopy in the field of inflammatory and infectious dermatoses opened a new horizon for scientific research. Above all, a new generation of young passionate researchers in the field has surfaced. Up to the year 2000, worldwide only 14 authors had published 10 or more papers on dermoscopy. Today, 145 authors have published more than 30 articles each.

The annual rate of published dermoscopy articles continued to increase steadily during the last decade. The 10 top authors of dermoscopy papers from 2011 to date are listed in Figure 4 and a list of the top 50 authors in Table 1.

In the year 2020, which was dominated by the COVID pandemic, the number of published dermoscopy papers reached a historic high of 2,253 items, with 118 different authors publishing more than 5 papers each. The top 10 are listed in Figure 5.

Several journals have published dermoscopy articles throughout the last decades, including all the top-ranking dermatology journals. The largest number has been published in the *Journal of the American Academy of Dermatology* (788), followed by the *Journal of the European Academy of Dermatology and Venereology* (571) (Figure 6). The fact that the official journals of the 2 largest dermatologic societies in the world published so many papers on the topic highlights their popularity among reader-clinicians. *Dermatology Practical and Conceptual* is not included in this list because it is a new journal that only recently has been indexed by Scopus. Being the official journal of the International Dermoscopy Society and given us the considerable space we need to devote to dermoscopy papers, and we are confident that our journal will one day appear high up in this list.

Predicting the future is a difficult task, and we cannot know if this trend will continue in the forthcoming years or if it will stabilize. What we believe to be true of the future is that dermoscopy will continue to be an invaluable tool for clinicians, inspire research, and unite the medical community. These are the ideals to which we aspire.

Aimilios Lallas, MD

Deputy Editor

Giuseppe Argenziano, MD

Editor-in-Chief

## Figures and Tables

**Figure 1:**
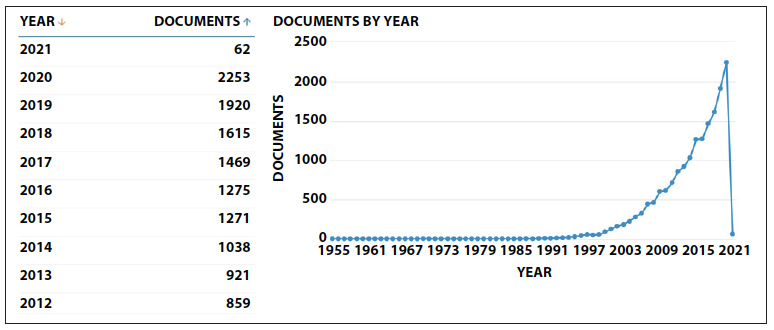


**Figure 2:**
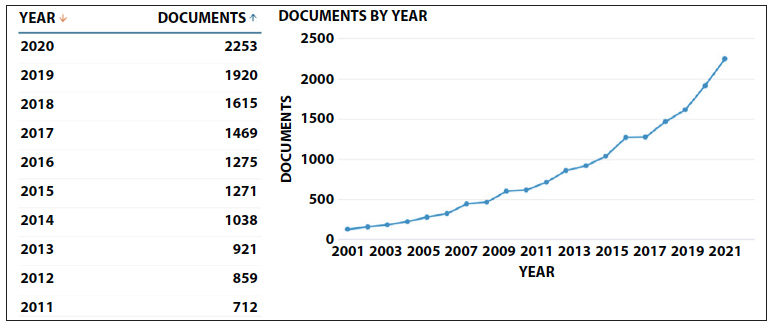


**Figure 3:**
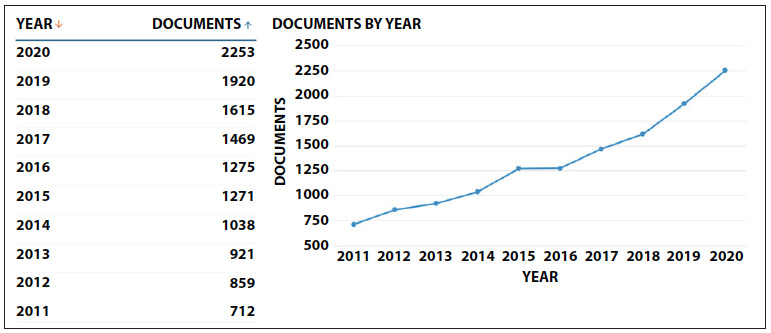


**Figure 4:**
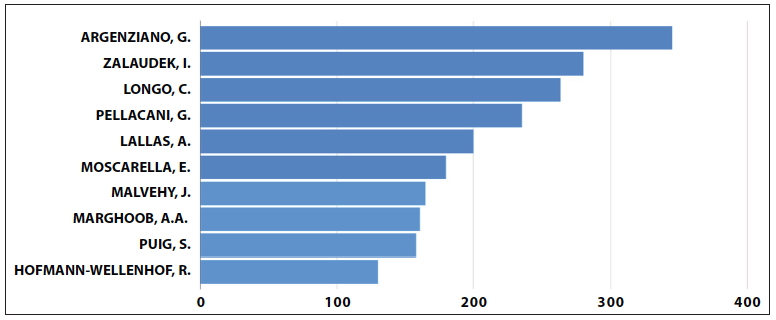


**Figure 5:**
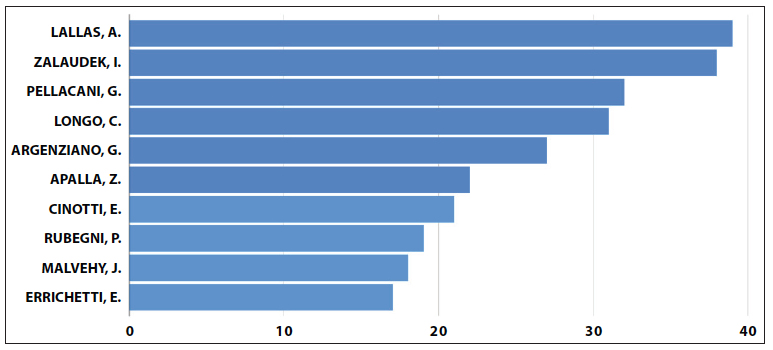


**Figure 6:**
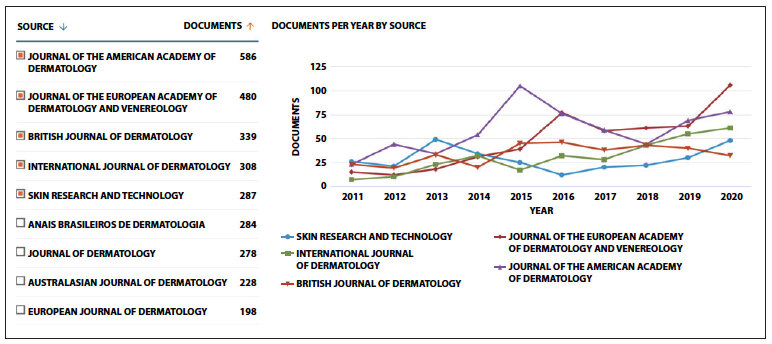


**Table 1:** 

Ranking	Author	Number of Papers
1	Argenziano, G.	345
2	Zalaudek, I.	280
3	Longo, C.	263
4	Pellacani, G.	235
5	Lallas, A.	200
6	Moscarella, E.	180
7	Malvehy, J.	165
8	Marghoob, A. A.	161
9	Puig, S.	158
10	Hofmann-Wellenhof, R.	130
11	Tosti, A.	120
12	Soyer, H. P.	116
13	Thomas, L.	111
14	Micali, G.	103
15	Cinotti, E.	102
16	Lacarrubba, F.	100
17	Carrera, C.	98
18	Piana, S.	88
19	Perrot, J. L.	85
20	Rubegni, P.	85
21	Kittler, H.	80
22	Apalla, Z.	79
23	Scope, A.	79
24	Patrizi, A.	77
25	Errichetti, E.	76
26	Piraccini, B. M.	68
27	Rudnicka, L.	68
28	Dika, E.	64
29	Haenssle, H. A.	61
30	Stinco, G.	61
31	Tanaka, M.	60
32	Farnetani, F.	59
33	Peris, K.	59
34	Dalle, S.	58
35	Bonifazi, E.	56
36	Dusza, S. W.	55
37	Labeille, B.	52
38	Halpern, A. C.	51
39	Mun, J.H.	50
40	Blum, A.	49
41	Tschandl, P.	49
42	Stanganelli, I.	48
43	Starace, M.	48
44	Verzì, A. E.	48
45	Braun, R.P.	47
46	Cambazard, F.	47
47	Piccolo, V.	47
48	Marchetti, M. A.	46
49	Kyrgidis, A.	44
50	Miteva, M.	44
